# Amphibians and reptiles of Parque Nacional da Serra das Lontras: an important center of endemism within the Atlantic Forest in southern Bahia, Brazil

**DOI:** 10.3897/zookeys.1002.53988

**Published:** 2020-12-10

**Authors:** Omar Rojas-Padilla, Vinícius Queiroz Menezes, Iuri Ribeiro Dias, Antônio Jorge Suzart Argôlo, Mirco Solé, Victor Goyannes Dill Orrico

**Affiliations:** 1 Laboratório de Herpetologia Tropical, Departamento de Ciências Biológicas, Universidade Estadual de Santa Cruz, Rodovia Jorge Amado, km 16, 45662-900, Ilhéus, Bahia, Brazil Universidade Estadual de Santa Cruz Ilhéus Brazil; 2 Laboratório de Sistemática de Vertebrados, Pontifícia Universidade Católica do Rio Grande do Sul, Av. Ipiranga, 6681, 90619-900, Porto Alegre, Rio Grande do Sul, Brazil Pontifícia Universidade Católica do Rio Grande do Sul Porto Alegre Brazil; 3 Herpetology Section, Zoologisches Forschungsmuseum Alexander Koenig, Adenauerallee 160, D-53113 Bonn, Germany Zoologisches Forschungsmuseum Alexander Koenig Bonn Germany

**Keywords:** Anura, Reptilia, Herpetofauna, biological inventory, species distribution

## Abstract

Information gaps about species distribution hamper the evaluation of conservation status and decisions on biodiversity conservation, affecting to a greater extent, areas with high species richness and endemism. In this context, biological inventories are an important tool to fill these gaps by providing data on the composition, richness, and abundance of species in each locality. The Parque Nacional da Serra das Lontras (PNSL) protects various mountain range just up 1000 m. in altitude, and, together with other conservation units, forms an ecological corridor in the southern part of the state of Bahia, within the Atlantic Forest hotspot. We conducted systematic samplings on transects, and opportunistic records in ponds and streams, in order to record amphibian and reptile species in the PNSL. We complement the sampling with the information available in the literature and in scientific collections. A total of 100 species (49 amphibians and 51 reptiles) was recorded, 53 of them endemic to the Atlantic Forest, 13 to the state of Bahia, and two known only from the PNSL. Hylidae was the most diverse family of amphibians (22 spp.) and Colubridae of reptiles (33 spp.). New information on the distribution and natural history of these species is provided, many of which have not yet been assessed by the IUCN while others have already been categorized as at risk of extinction at the regional level. Results confirm the high species richness and rates of endemism in southern Bahia and highlight the importance of protecting high altitude areas for the preservation of evolutionary and ecological processes within the Atlantic Forest.

## Introduction

Biodiversity inventories are crucial in megadiverse countries, particularly in those that still have areas that are poorly sampled or without information about the species that inhabit them (Trindade-Filho et al. 2012; Verdade et al. 2012). These inventories provide data on natural history, behavior and make it possible to find taxa previously unknown to the region or still undescribed (Verdade et al. 2012; Oliveira et al. 2017). At the same time, they provide updated data on the state of conservation of the locality sampled and the threats present for the reported populations.

Deforestation, climate change, pollution, invasive species and diseases are among the main threats to biodiversity (Lips et al. 2005; Butchart et al. 2010). According to IUCN (International Union for Conservation of Nature), 41% of amphibian species and 22% of reptiles are included in some threat category (Hoffmann et al. 2010). In fact, many species of reptiles still lack enough information to allow their categorization (Böhm et al. 2013) making it even more difficult to implement effective actions for their conservation.

The Atlantic Forest biome stands out for having a high species richness and endemism rate. Despite harboring species not yet described and discovered (Morellato and Haddad 2000), it is estimated that it houses half of the endangered species of Brazil, 38.5% of which are endemic to this biome (ICMBio 2018a). However, the biome has also shown high rates of deforestation and is considered one of the biodiversity hotspots in the world (Myers et al. 2000). The south of the state of Bahia, located in Northeastern Brazil, is still home to the largest forest remnants of the Atlantic Forest in this part of the country, most of them associated with slopes or altitude zones (Thomas et al. 1998; Oliveira-Filho and Fontes 2000; Amorim et al. 2009). In these zones, high levels of plant richness and endemism (Amorim et al. 2009) and the second largest number of amphibian species for the entire biome have been recorded (Dias et al. 2014).

The Parque Nacional da Serra das Lontras (PNSL), together with two more conservation units, the Refúgio de Vida Silvestre Una and the Reserva Biológica Una, form an ecological corridor which protects from low areas of the Atlantic coast to mountain peaks of just over 1000 m. in altitude. From the PNSL the presence of 709 species of angiosperms has been documented, the largest number of species reported for an altitude area in southern Bahia (Amorim et al. 2009). Also, 295 species of birds have already been recorded, 18 of them threatened with extinction (Silveira et al. 2005). For amphibians, 16 species were reported (Silvano and Pimenta 2003). However, the sampling effort was very low and there is no list of reptiles available for the region. Even so, new species of birds, amphibians and reptiles have been described with material collect in the PNSL (see Pacheco et al. 1996; Recorder et al. 2010; Teixeira et al. 2013). In order to provide information that can help in the elaboration of species management plans, conservation plans and aid the categorization of species, we complement and update the list of amphibians and present, for the first time, a list of reptiles for this conservation unit.

## Materials and methods

### Study area

**Figure 1.**
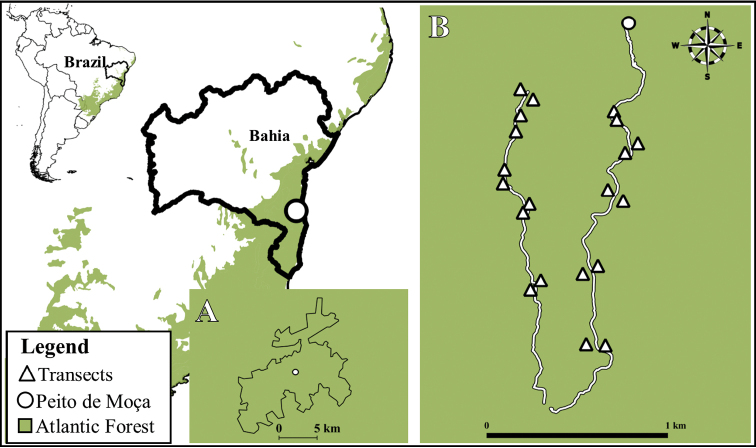
Location of the Parque Nacional da Serra das Lontras and the evaluated transects. **A** The Parque Nacional da Serra das Lontras **B** trails and transects sampled during 2017 and 2018.

The PNSL (Fig. 1) is a federal conservation unit located in the municipalities of Arataca and Una, in the southern region of Bahia, Brazil (15.16979°S, 39.35047°W). It is located 56 km away from Ilhéus and 265 km from Salvador, the state capital and has an extension of 113.43 km^2^ with an altitudinal gradient from 300 to just over 1000 m. of altitude. The climate is classified as equatorial rainforest, fully humid (Af) (Kottek et al. 2006).

**Figure 2.**
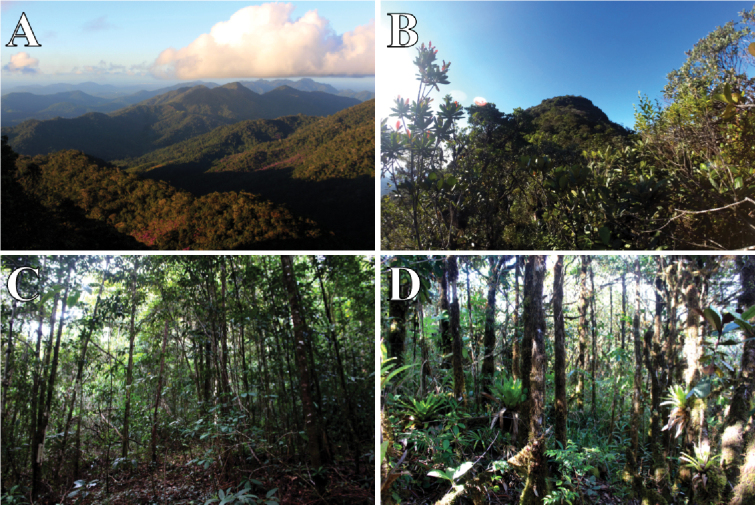
General and detail view of the change of vegetation in the Parque Nacional da Serra das Lontras. **A** Panoramic view from “Peito de Moça” (930 m altitude) **B** view of the “Peito de Moça” **C** primary vegetation with thin and tall trees with closed canopy below 750–800 m altitude **D** smaller vegetation with epiphytes and canopy more open in the peaks.

The vegetation of the PNSL is formed by a mosaic of forest cover, with predominance of primary and late secondary forests, areas in recovery and areas of “cabruca” (cocoa crops shaded by native trees). The altitude gradient facilitates the presence of different plant formations, where thin tall trees with a closed canopy and shrubby vegetation predominate up to 750–800 m altitude, and smaller trees with epiphytes and a more open canopy dominate in higher altitudes (Fig. 2).

### Data collection

We used the following methodologies for the sampling of the herpetofauna in the PNSL: i) visual and acoustic active search in transects in the forest (Heyer et al. 1994), ii) active search in water bodies: streams, temporary and permanent ponds (Heyer et al. 1994), iii) opportunistic records during our displacement, and iv) review of material deposited in the Museu de Zoologia of the Universidade Estadual de Santa Cruz. To complement the list of recorded species, we included the records of other studies carried out in the PNSL (Silvano and Pimenta 2003; Recoder et al. 2010; Teixeira et al. 2013).

Fieldwork was carried out during 44 sampling days during seven sampling campaigns: December 9–11 2014; March 9 and 10 2015; October 23–26 2017; and February 19–29, March 6–12, October 8–15, and December 10–18 in 2018.

In the years 2014 and 2015 we sampled 14 transects of 50 meters in length, localized between 700 and 900 m of altitude inside the primary forest. Each transect was sampled by two researchers only once for 40 minutes, totaling a sampling effort of 9.3 man hours. This sampling was complemented with active non-standard searches in streams and temporary ponds inside the forest.

In 2017, we conducted non-standardized searches in the interior of the forest during the opening of trails and definition of places for the installation of complementary transects. Active searches without time limits were also carried out in streams and ponds.

In 2018, we installed two new 50 m long transects in each of the following altitudes: 450, 550, 650, 750, and 850 m in two mountains. Ten transects were installed on each mountain, totaling 20 transects. Each was sampled for 50 minutes by two researchers only once per campaign. In this period, each transect was evaluated three times, adding up to a sampling effort of 50 man hours. By the end of the study, we completed 59.30 man hours of sampling in the PNSL.

For the nomenclature of amphibian species, we follow Frost (2020). Regarding *Adelophryne* spp. we follow Lourenço-de-Moraes et al. (2018), and for *Adenomera* we follow Fouquet et al. (2014). For reptiles we follow Uetz and Hošek (2020); and for the particulary case of *Thamnodynastes*, we follow the sugestions by Franco and Ferreira (2002). We identified the endemic species of the Atlantic Forests and for Bahia state. Each recorded species was identified according to the proposals made for the biome by Rossa-Feres et al. (2017) and Tozetti et al. (2017) for amphibians and reptiles, respectively. Regarding the state; we revised the distribution sections in Frost (2020) for the amphibians, and the detailed list provided by Costa and Bérnils (2018) for reptiles.

### Sampling of specimens and conservation status

All individuals collected in this work were covered by a license issued by the Instituto Chico Mendes de Conservação da Biodiversidade (ICMBio 59889-1) and they were deposited in the herpetological collection of the Museu de Zoologia of the Universidade Estadual de Santa Cruz (**MZUESC**) in Ilhéus, Bahia, Brazil. We identified the conservation status of each species at the state, federal and international scale using reference lists from the Secretaria de Meio Ambiente do Estado da Bahia – SEMA (2017), from the Instituto Chico Mendes de Conservação da Biodiversidade – ICMBio (2018b, 2018c), and the IUCN (2019). The SEMA and ICMBio list provide only the categorization of species considered to be at risk of extinction. The categories of the identified species are as follows: DD, data deficient; LC, Least Concern; NT, Near Threatened; VU, Vulnerable; and EN, Endangered.

## Results

**Figure 3.**
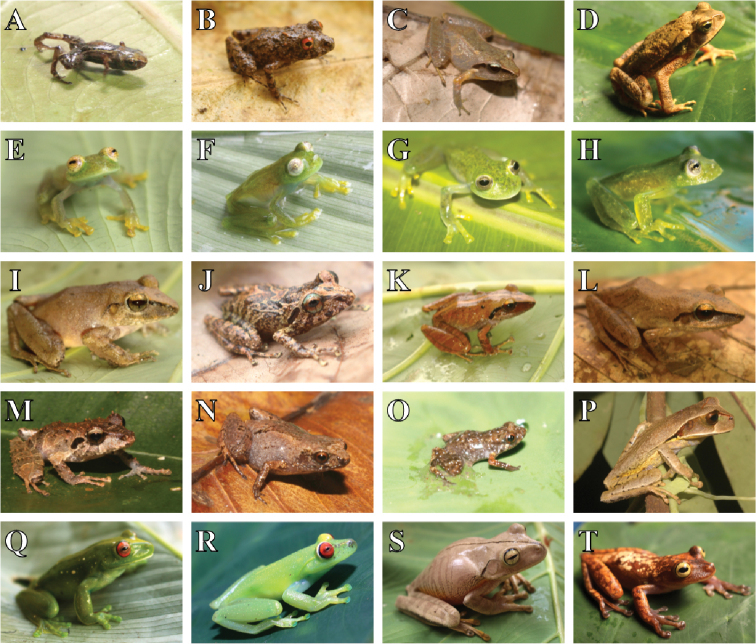
Amphibians recorded in the Parque Nacional da Serra das Lontras: **A***Brachycephalus pulex***B***Ischnocnema verrucosa***C**Ischnocnema cf. parva**D***Rhinella crucifer***E***Vitreorana baliomma***F***V. eurygnatha***G***Vitreorana* sp.nov. **H***V. uranoscopa***I***Haddadus binotatus***J***Pristimantis* sp. 1 **K***Pristimantis* sp. 2 **L***Pristimantis paulodutrai***M***Pristimantis vinhai***N***Adelophryne* sp. 8 **O***Adelophryne* sp. 2 **P***Gastrotheca recava***Q***Aplastodiscus ibirapitanga***R***A. weygoldti***S***Boana faber***T***Bokermannohyla lucianae*.

**Figure 4.**
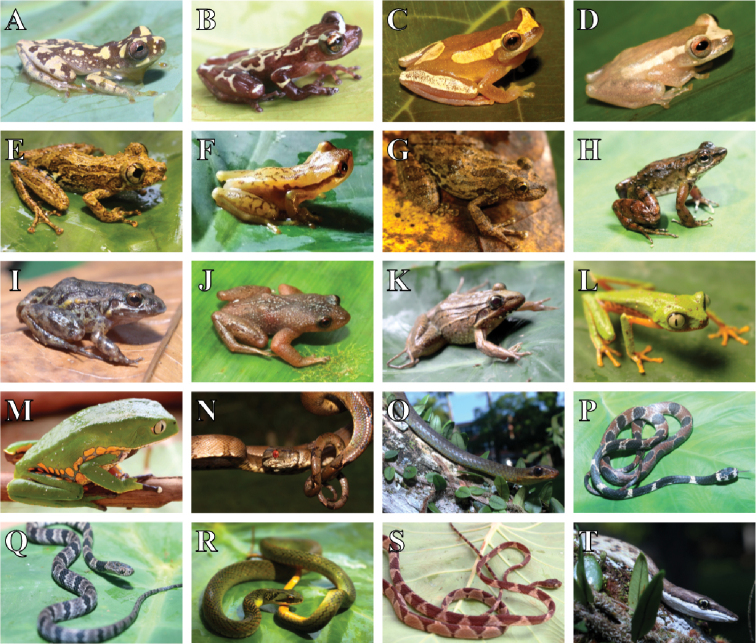
Amphibians and reptiles recorded in the Parque Nacional da Serra das Lontras. **A***Dendropsophus branneri***B**Dendropsophus aff. bromeliaceus**C***D. elegans***D***D. haddadi***E***Ololygon strigilata***F***Phyllodytes* sp. 1 **G**Scinax cf. x-signatus**H***Crossodactylus* sp. **I***Adenomera* clade M **J***Crossodactylodes septentrionalis***K**Leptodactylus cf. latrans**L***Phasmahyla spectabilis***M***Phyllomedusa burmeisteri***N***Corallus hortulanus***O***Chironius fuscus***P***Dipsas catesbyi***Q***Dipsas neuwiedi***R***Erythrolamprus reginae*, **S***Imantodes cenchoa***T***Oxybelis aeneus*.

**Figure 5.**
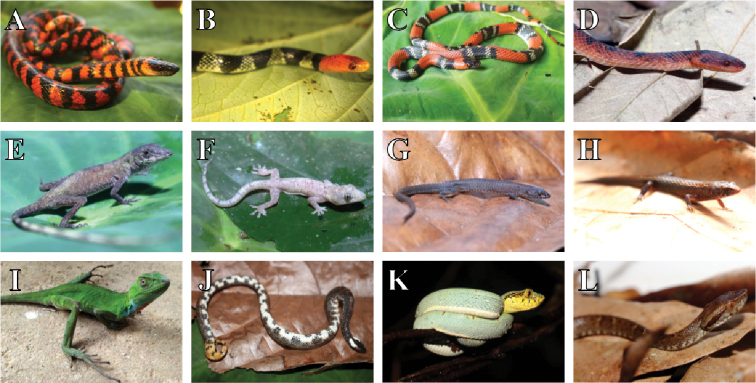
Reptiles recorded in the Parque Nacional da Serra das Lontras. **A***Oxyrhopus clathratus***B***O. formosus***C***O. guibei***D***Xenopholis scalaris***E***Anolis fuscoauratus***F***Hemidactylus mabouia***G***Leposoma nanodactylus***H***L. scincoides***I***Enyalius catenatus***J***Tropidophis grapiuna***K***Bothrops bilineatus***L***B. jararaca*.

We recorded 100 species, 49 of amphibians, and 51 of reptiles in the PNSL (Table 1, Figs 3–5). Ten families of amphibians, being the most diverse Hylidae (22 spp.), followed by Craugastoridae (06 spp.), Centrolenidae and Bufonidae (04 spp. each), Brachycephalidae and Leptodactylidae (03 spp. each), Eleutherodactylidae, Phyllomedusidae and Hemiphractidae (02 spp. each), and Hylodidae (01 sp.). In turn, we report 13 families of reptiles: Colubridae (33 spp.), Viperidae (04 spp.), Amphisbaenidae, Boidae, and Gymnophthalmidae (02 spp. each), and a species each of the families Chelidae, Dactyloidae, Elapidae, Gekkonidae, Leiosauridae, Polychrotidae, Teiidae, and Tropidophiidae.

Forty amphibians and 13 reptiles are endemic of the Atlantic Forest biome. Of these, eleven species of anurans and two of reptiles are restricted to the state of Bahia; and two anurans, *Dendrophryniscus oreites* and *Crossodactylus septentrionalis*, to the PNSL (Table 1). Although some individuals of amphibian are identified as “sp.”, "cf.", or “aff.”, individuals of the genus *Phyllodytes* are being considered endemic to the biome, as, until now, they have not been reported from other biomes.

**Table 1.** Amphibians and reptiles in the Parque Nacional da Serra das Lontras, Bahia, Brazil. Key: **C.S.**–Conservation Status, DD: Data Deficient, LC: Least Concern; EN: Endangered, VU: Vulnerable, according: 1: Secretaria de Meio Ambiente – Bahia state, 2: Instituto Chico Mendes de Biodiversidade, 3: International Union for Conservation of Nature. **EN.**– endemism, AF: Atlantic Forest, BA: Bahia. **S.M**– sampling method, AS: Active search, Tr: visual and acoustic active search in transects, Op: opportunistic records, Bi: bibliographic revision, Mu: individuals deposited in the herpetological collection of the Museu de Zoologia of the Universidade Estadual de Santa Cruz.

Class / Order / Family / Species	C.S.	EN.	S.M.
** Amphibia **			
** Anura **			
** Brachycephalidae **			
*Brachycephalus pulex* Napoli, Caramaschi, Cruz & Dias, 2011		AF, BA	Tr, AS, Mu
*Ischnocnema verrucosa* (Reinhardt & Lütken, 1862)	EN ^1^	AF	AS
Ischnocnema cf. parva		AF	Tr, AS
** Bufonidae **			
*Dendrophryniscus oreites* Recoder, Teixeira, Cassimiro, Camacho & Rodrigues, 2010		AF, BA	Bi
*Dendrophryniscus proboscideus* (Boulenger, 1882)	DD ^3^	AF	AS, Bi
*Rhinella crucifer* (Wied-Neuwied, 1821)	LC ^3^	AF	Op, Mu, Bi
*Rhinella hoogmoedi* Caramaschi & Pombal, 2006	LC ^3^	AF	Op, Bi
** Centrolenidae **			
*Vitreorana baliomma* Pontes, Caramaschi & Pombal, 2014		AF	AS
*Vitreorana eurygnatha* (Lutz, 1925)	EN^1^, LC^3^		Tr, AS
*Vitreorana* sp. nov.		AF	AS
*Vitreorana uranoscopa* (Müller, 1924)	LC ^3^	AF	AS, Mu
** Craugastoridae **			
“*Eleutherodactylus*” *bilineatus* (Bokermann, 1975)	LC ^3^	AF, BA	Op
*Haddadus binotatus* (Spix, 1824)	LC ^3^	AF	Tr, Op, Bi
*Pristimantis* sp. 1			Tr, AS
*Pristimantis* sp. 2			Tr, AS
*Pristimantis paulodutrai* (Bokermann, 1975)	LC ^3^	AF, BA	Tr, AS
*Pristimantis vinhai* (Bokermann, 1975)	LC ^3^	AF, BA	Tr, Op, Mu, Bi
** Eleutherodactylidae **			
*Adelophryne* sp. 2 (sensu Lourenço-de-Moraes et al. 2018)		AF	Tr, AS
*Adelophryne* sp. 8 (sensu Lourenço-de-Moraes et al. 2018)		AF	Tr, AS
** Hemiphractidae **			
*Gastrotheca pulchra* Caramaschi & Rodrigues, 2007		AF	Op
*Gastrotheca recava* Teixeira, Vechio, Recoder, Carnaval, Strangas, Damasceno, Sena & Rodrigues, 2012		AF, BA	Tr, AS, Op
** Hylidae **			
*Aplastodiscus ibirapitanga* (Cruz, Pimenta & Silvano, 2003)	LC ^3^	AF	Op
*Aplastodiscus weygoldti* (Cruz & Peixoto, 1987)	NT ^3^	AF	Tr, Op
*Boana albomarginata* (Spix, 1824)	LC ^3^	AF	Op
*Boana crepitans* (Wied-Neuwied, 1824)			Bi, Mu
*Boana exastis* (Caramaschi & Rodrigues, 2003)	DD ^3^	AF	AS
*Boana faber* (Wied-Neuwied, 1821)	LC ^3^		Op, Bi
*Boana pombali* (Caramaschi, Pimenta & Feio, 2004)	LC ^3^	AF	Op
*Bokermannohyla lucianae* (Napoli & Pimenta, 2003)	DD ^3^	AF, BA	Tr, Op, Bi
*Dendropsophus bipunctatus* (Spix, 1824)	LC ^3^	AF	Bi
*Dendropsophus branneri* (Cochran, 1948)	LC ^3^	AF	AS
Dendropsophus aff. bromeliaceus		AF	Tr
*Dendropsophus elegans* (Wied-Neuwied, 1824)	LC ^3^	AF	AS, Bi
*Dendropsophus haddadi* (Bastos & Pombal, 1996)	LC ^3^	AF	AS
*Dendropsophus minutus* (Peters, 1872)	LC ^3^		Bi
*Ololygon strigilata* (Spix, 1824)	DD ^3^	AF, BA	Op, Bi
Phyllodytes cf. maculosus		AF	Tr
*Phyllodytes* sp. 1		AF	Tr, AS, Op
*Phyllodytes* sp. 2		AF	Tr, AS, Op
*Phyllodytes megatympanum* Marciano, Lantyer-Silva & Solé, 2017		AF, BA	Tr, AS
*Scinax juncae* Nunes & Pombal, 2010		AF, BA	AS, Bi
*Scinax eurydice* (Bokermann, 1968)	LC ^3^	AF	Mu
Scinax cf. x-signatus			AS, Op, Bi
** Hylodidae **			
*Crossodactylus* sp.			AS
** Leptodactylidae **			
*Adenomera* clade M (sensu Fouquet et al. 2014)		AF	AS
*Crossodactylodes septentrionalis* Teixeira, Recoder, Amaro, Damasceno, Cassimiro & Rodrigues, 2013		AF, BA	AS
Leptodactylus cf. latrans			Op, Bi
** Phyllomedusidae **			
*Phasmahyla spectabilis* Cruz, Feio & Nascimento, 2008	VU^1^, DD^3^	AF	Op
*Phyllomedusa burmeisteri* Boulenger, 1882	LC ^3^	AF	Tr, Bi
** Reptilia **			
** Testudines **			
** Chelidae **			
*Hydromedusa maximiliani* (Mikan, 1820)	EN^1^, VU^3^	AF	Mu
** Squamata **			
** Amphisbaenidae **			
*Amphisbaena pretrei* Duméril & Bibron, 1839	LC ^3^		Mu
*Leposternon* sp.			Mu
** Boidae **			
*Corallus hortulanus* (Linnaeus, 1758)	LC ^3^		Op, Mu
*Epicrates cenchria* (Linnaeus, 1758)			Mu
** Colubridae **			
*Cercophis auratus* (Schlegel, 1837)	VU^1^, DD^3^		Mu
*Chironius exoletus* (Linnaeus, 1758)			Mu
*Chironius foveatus* Bailey, 1955	LC ^3^	AF	Mu
*Chironius fuscus* (Linnaeus, 1758)			Tr, Op, Mu
*Chironius laevicollis* (Wied-Neuwied, 1824)	LC ^3^	AF	Mu
*Coronelaps lepidus* (Reinhardt, 1861)	LC ^3^	AF	Mu
*Dipsas catesbyi* (Sentzen, 1796)	LC ^3^		AS, Mu
*Dipsas indica* Laurenti, 1768			Mu
*Dipsas neuwiedi* (Ihering, 1911)	LC ^3^	AF	AS, Mu
*Dipsas variegata* (Duméril, Bibron & Duméril, 1854)			Mu
*Drymoluber dichrous* (Peters, 1863)	LC ^3^		Mu
*Echinanthera cephalostriata* Di Bernardo, 1996	LC ^3^	AF	Mu
*Elapomorphus wuchereri* Günther, 1861		AF	Mu
*Erythrolamprus aesculapii* (Linnaeus, 1758)			Mu
*Erythrolamprus miliaris* (Linnaeus, 1758)	LC ^3^		Mu
*Erythrolamprus poecilogyrus* (Wied-Neuwied, 1825)			Mu
*Erythrolamprus reginae* (Linnaeus, 1758)			Op, Mu
*Erythrolamprus taeniogaster* (Jan, 1863)	LC ^3^		Mu
*Imantodes cenchoa* (Linnaeus, 1758)	LC ^3^		Tr, Mu
*Leptodeira annulata* (Linnaeus, 1758)	LC ^3^		Mu
*Oxybelis aeneus* (Wagler, 1824)			Op, Mu
*Oxyrhopus clathratus* Duméril, Bibron & Duméril, 1854	VU ^1^		Op
*Oxyrhopus formosus* (Wied-Neuwied, 1829)	EN ^1^		Op, Mu
*Oxyrhopus guibei* Hoge & Romano, 1977	LC ^3^		Tr, Mu
*Oxyrhopus petolarius* (Linnaeus, 1758)			Mu
*Philodryas olfersii* (Lichtenstein, 1823)			Mu
*Pseudoboa nigra* (Duméril, Bibron & Duméril, 1854)			Mu
*Siphlophis compressus* (Daudin, 1803)	LC ^3^		Mu
*Spilotes pullatus* Linnaeus, 1758			Op, Mu
*Spilotes sulphureus* (Wagler, 1824)			Mu
Thamnodynastes cf. nattereri (Mikan, 1828)	LC ^3^		AS, Mu
*Xenodon rabdocephalus* (Wied-Neuwied, 1824)			Mu
*Xenopholis scalaris* (Wucherer, 1861)	LC ^3^		Op
** Dactyloidae **			
*Anolis fuscoauratus* D’Orbigny, 1837			Tr, Op
** Elapidae **			
*Micrurus corallinus* (Merrem, 1820)		AF	Mu
** Gekkonidae **			
*Hemidactylus mabouia* (Moreau de Jonnès, 1818)			Op
** Gymnophthalmidae **			
*Leposoma nanodactylus* Rodrigues, 1997	EN^1^,^2^	AF, BA	Tr, AS
*Leposoma scincoides* Spix, 1825		AF	Op
** Leiosauridae **			
*Enyalius catenatus* (Wied-Neuwied, 1821)	LC ^3^	AF	Tr, Op, Mu
** Polychrotidae **			
*Polychrus marmoratus* (Linnaeus, 1758)	LC ^3^		Mu
** Teiidae **			
*Ameiva ameiva* (Linnaeus, 1758)			Mu
** Tropidophiidae **			
*Tropidophis grapiuna* Curcio, Nunes, Argôlo, Skuk & Rodrigues, 2012	EN^1^, VU^2^	AF, BA	Tr
** Viperidae **			
*Bothrops bilineatus* (Wied-Neuwied, 1821)	VU ^1^		Op, Mu
*Bothrops jararaca* (Wied-Neuwied, 1824)		AF	Tr, Op, Mu
*Bothrops leucurus* Wagler, 1824			Mu
*Lachesis muta* (Linnaeus, 1766)	VU ^1^		Mu

### Conservation status

According to SEMA (2017), six of our recorded species are considered endangered at state level: *Ischnocnema verrucosa*, *Oxyrhopus formosus*, *Tropidophis grapiuna*, and *Vitreorana eurygnatha* are categorized as EN, and *O. clathratus* and *Phasmahyla spectabilis* as VU. At federal level, according to ICMBio (2018b, c) *Leposoma nanodactylus* is categorized as EN, and *T. grapiuna* as VU. On the other hand, according to IUCN, *Bokermannohyla lucianae* and *P. spectabilis* are considered as DD, *Aplastodiscus weygoldti* as NT, and other 18 species as LC. However, 42 of the recorded species have not been categorized by IUCN (Table 1).

## Discussion

Brazil is currently home to 1137 species of amphibians and 795 reptiles (Costa and Bérnils 2018; Segalla et al. 2019). However, new species are constantly being described from different biomes (Ferrão et al. 2017; Orrico et al. 2017; Vörös et al. 2017; Arias et al. 2018; among others), reflecting our scant knowledge about the species richness of these groups. From the state of Bahia, approximately 190 species of amphibians and 278 reptiles with ca. 129 species of snakes (Hamdan and Lira-da-Silva 2012; Dias et al. 2014; Costa and Bérnils 2018) have been reported so far. Here we report 49% of the total amphibian species and 19% of reptiles known for the state from an area slightly larger than 110 km². We believe that this number does not reflect the real diversity of amphibians and reptiles in the PNSL.

The first amphibian inventory undertaken at PNSL recorded 16 species (Silvano and Pimenta 2003). Due to taxonomic changes in different groups after that publication, we updated the binomial names and discuss some of the identifications. In order to avoid under- or overestimation of species richness, we assign the names to the species that were also found in our samples and hypothesize the presence of other species based on other records in nearby areas.

Species of *Bufo* were transferred to the genus *Rhinella* (Frost et al. 2006). *Rhamphophryne proboscidea* is now included in *Dendrophryniscus* (Fouquet et al. 2012a); we did not record this species, but its presence was confirmed in the last revision of the genus (see Cruz et al. 2019) and has also been reported in nearby areas (Silva et al. 2011). The ancient specious genus *Eleutherodactylus* was revised and several of its species have been transferred to other genera, thus *E. binotatus* moved to *Haddadus* (Hedges et al. 2008), and *E. vinhai* first to *Ischnocnema* (Heinicke et al. 2007, Hedges et al. 2008) and later to *Pristimantis* (Canedo and Haddad 2012). Likewise, the six reported species of *Hyla* currently belong to the following binomials: *Boana crepitans*, *B. faber*, *Bokermannohyla lucianae*, *Dendropsophus bipunctatus*, *D. elegans*, and *D. minutus* (Faivovich et al. 2005; Dubois 2017). We note that the record of *Bokermannohyla lucianae* was identified as “*Hyla* sp. n3” (Silvano and Pimenta 2003), with the species being described a year later (see Napoli and Pimenta 2004). We consider the record of *Scinax cuspidatus* as *S. juncae* because we recorded several individuals vocalizing in a pond. In the same way, the record of *S. fuscovarius* is now attributed to S. cf. x-signatus. Finally, we relate *Leptodactylus ocellatus* to L. cf. latrans, given that there are species delimitation problems, being barely distinguishable from the species complex including *L. chaquensis* and *L. macrosternum* (de Sá et al. 2014).

Dias et al. (2014) carried out an amphibian inventory in an area close to the PNSL, the RPPN Serra Bonita (SB), where they found 80 species. The SB, in addition to being close the PNSL (31.15 km away as a straight line), it shares the same relief characteristics (200–950 m) and vegetation types (Amorim et al. 2009). Our research differs from that developed by Dias et al. (2014) regarding the sampling effort (192 man hours in transects in the forest, versus 59.3 man hours in PNSL), installation of transects close to streams, and installation of pitfall traps. Although we sampled for several days in the rainy season (approximately one week), the presence of seasonal ponds was limited and, when formed, the number of species with expected explosive reproduction were not found (Duellman and Trueb 1994; Wells 2008). We also highlight that the area sampled in the PNSL represents only a small fraction of the park’s extension.

We found 49 species of amphibians that represent more than half of those known from SB, an area considered to harbor the second largest species richness in the Atlantic Forest (Dias et al. 2014). PNSL and SB share 31 species of anurans. We believe that with more sampling efforts in streams, temporary and permanent ponds, and in other areas of the PNSL, we would find several of the species already reported from SB: *Boana semilineata*, *Bokermannohyla circumdata*, *Ceratophrys aurita*, *Chiasmocleis crucis*, *Dendropsophus anceps*, *D. giesleri*, *D. oliverai*, *Leptodactylus cupreus*, *L. mystaceus*, *Physalaemus camacan*, *P. erikae*, *Pipa carvalhoi*, *Pithecopus rhodei*, *Proceratophrys renalis*, *Pr. schirchi*, *Rhinella granulosa*, *R. jimi*, *Ololygon argyreonata*, *Siphonops annulatus*, *Sphaenorhynchus prasinus*, *Stereocyclops histrio*, *S. incrassatus*, and *Trachycephalus mesophaeus* which would increase our list by another 24 species. However, in the PNSL we have recorded four species not yet reported from the SB, Dendropsophus cf. bromeliaceus, *Gastrotheca recava*, *Vitreorana baliomma*, and *Vitreorana* sp. nov.

Considering the taxonomic uncertainties and the possibility of undescribed entities in the region, we try to assign identifications to the finest possible level. *Pristimantis* sp. 1 differs from all other species of *Pristimantis* found in the PNSL by its eye color, spotted dorsal pattern, and call parameters. *Pristimantis* sp. 2 is the same species reported as *Pristimantis* sp. from the Reserva Ecológica Michelin (Mira-Mendes et al. 2018). Fouquet et al. (2012b) defined *Adelophryne* populations from neighboring areas as *A. pachydactyla* but further research refuted this hypothesis (see Dominato et al. 2018; Lourenço-de-Moraes et al. 2018). In our sampling we found two species of this genus and due to their morphological characteristics, we identified them as *Adelophryne* sp. 2 and *Adelophryne* sp. 8 sensu Lourenço-de-Moraes et al. (2018). Likewise, individuals from *Adenomera* are attributed to clade M, sensu Fouquet et al. (2014).

The flea-toad, *Brachycephalus pulex*, was known only from the upper parts of the type locality in Serra Bonita (Napoli et al. 2011). Our record expands its distribution by 31 km in a straight line. *Bokermannohyla lucianae* appears to have a distribution bounded by the Cachoeira and Jequitinhonha rivers in the southern part of Bahia (Dias et al. 2011), with PNSL being only the fourth known location for the species. *Pristimantis* sp. 2 is distributed in lowland forest of southern Bahia (Mira-Mendes et al. 2018).

Five species of the genus *Vitreorana* are known from the Atlantic Forest biome (Rossa-Feres et al. 2017). Although Rossa-Feres et al. (2017) considered *V. eurygnatha* as endemic to the Atlantic Forest, the species was reported in a locality within the Cerrado biome (Cintra et al. 2013). However, the PNSL, with four syntopic species (*V. baliomma*, *V. eurygnatha*, *V. uranoscopa*, and one species as yet undescribed) is the most diverse site for the genus in the Atlantic Forest, where usually only one or two species are found (see Pontes et al. 2014; Dias et al. 2014; Mira-Mendes et al. 2018). We heard vocalizations of *V. eurygnatha* and *V. uranoscopa* in the months of February and April, and *V. baliomma* only in April, all records being made in 2018. All these species use the vegetation on the banks of streams to vocalize, mate, and for oviposition (Haga et al. 2014; Zaracho 2014), with *V. baliomma* and *V. eurygnatha* sharing vocalization microhabitats. The new species of *Vitreorana* differs from the others by morphological and genetic characters.

Most of the reptile’s records were obtained from material deposited at MZUESC. During our systematic sampling, we did not install pitfall traps, which could have increased the number of lizards and snakes of terrestrial and fossorial habitats in our records (Cechin and Martins 2000). At the same time, the fact that our samplings were carried out mainly at night may have privileged the record of amphibian species (Doan 2003). We emphasize that, in the methodological evaluations, eleven species were recorded by a single individual. In absolute numbers, the PNSL can be considered as the third locality with the greatest reptile richness in the state of Bahia, being only surpassed by the Serra da Jibóia and the Serra do Timbó, with 59 and 54 species, respectively (vs. 51 from PNSL) (Freitas et al. 2018; Freitas et al. 2019).

The rare turtle *Hydromedusa maximiliani* has records associated to water bodies within primary forests in mountainous regions, with previous records from other localities in Bahia (Argôlo and Freitas 2002). Although Tozetti et al. (2017) considered *Oxyrhopus formosus* to be endemic to the Atlantic Forest, its distribution is unclear with records scattered through the Brazilian, Ecuadorian, and Peruvian Amazon (Catenazzi et al. 2013; Wallach et al. 2014; Costa and Bérnils 2018). This taxon is considered a species complex with populations in Guyana, Colombia, and some places in Ecuador having been reidentified as *O. occipitalis* (Lynch 2009; MacCulloch et al. 2009). In the Atlantic Forest, *O. formosus* is considered a rare species categorized as EN in the state of Bahia (Argôlo 2004; SEMA 2017), and reported from four localities within this biome: Almadina and Coaraci (Argôlo et al. 2012; Dias et al. 2014b) and Mucuri, the type locality (sensu Vanzolini and Myers 2015), all in the state of Bahia; and Duas Barras in Espírito Santo state (Tonini et al. 2010). Considering the conservation status and doubts about its geographical distribution, molecular, pholidosic, and other morphological data can help solve the taxonomic problem of this species with disjunct distribution.

*Oxyrhopus clathratus* inhabits dense coastal ombrophilous and mixed ombrophilous forests from the northeast and southeast of Brazil (Tozetti et al. 2017), and reaches the north of Argentina (Di-Bernardo et al. 2012). Di-Bernardo et al. (2012) suggested that the color patterns of individuals are related to altitude, and the pattern of our individual is consistent with the one most common in lowland areas, although found at ~750 m. Our record represents the third for Bahia, having previously been found in Barra do Choça (Argôlo 2001) and in the SB (Medeiros et al. 2010).

Only two individuals of *Tropidophis grapiuna* are known in the literature, both collected in ombrophilous forest between 725–750 m altitude in the southern portion of Bahia (Curcio et al. 2012). Since its description, no other individuals have been collected. We found an individual in the leaf litter at 550 m, representing the first collected male, the lowest altitudinal record, and the first record inside a conservation area for this species.

The species *Cercophis auratus*, *Echinanthera cephalostriata*, *Hydromedusa maximiliani*, *Oxyrhopus clathratus*, and *Tropidophis grapiuna* represent populations restricted to montane forests in the latitude range of this study (Argôlo and Freitas 2002; Argôlo 2009). In fact, long-term sampling in southern Bahia has never detected any of these species in the lowlands of the region (Argôlo 2004). The lizards *Leposoma nanodactylus* and *L. puk* are known principally from mountain forests of southern Bahia. *Leposoma nanodactylus* has records in the PNSL and, in view of the known distribution of *L. puk* (Rodrigues et al. 2002; Rodrigues et al. 2013), it is likely that this species also occurs there. This information helps to highlight the importance of the PNSL for biodiversity conservation.

Of the 100 species reported in the PNSL, 53 are endemic to the Atlantic Forest and 13 of these are endemic to the state of Bahia, of which only two, *Crossodactylodes septentrionalis* and *Dendrophryniscus oreites*, are, until now, restricted to the park. One of the theories to explain the large number of endemic species in this biome is that of the Pleistocene refuge hypothesis (Haffer 1997). The PNSL is located inside the “Refúgio da Bahia”, identified as the one with the greatest extension in the biome, a zone of climatic stability that allowed the maintenance of different species during the last glacial maximum (Carnaval et al. 2009). In this way, the altitude areas of the region may have functioned as opportune places of climatic stability and, subsequently allowed a diversification of the surviving fauna (Graham et al. 2014).

Climatic conditions in these areas can shape the lives of the amphibians and reptiles that inhabit them (Duellman and Trueb 1994). It has been proposed that small frogs of the genus *Brachycephalus* inhabit areas of altitude due to a dependence on temperature and microclimate that are modulated by mist (Haddad et al. 2008). The scarcity of water bodies in the higher parts of the mountains may have favored these places to be occupied by species of genera with direct development, such as *Adelophryne*, *Brachycephalus*, *Ischnocnema*, and *Pristimantis* (Siqueira and Rocha 2013), and those using bromeliads for tadpole development, *Crossodactylodes* spp. and *Phyllodytes* spp. (Sabagh et al. 2017). In fact, we found species of these genera in the highest locals of the PNSL where bromeliads are more abundant.

Lastly, the expansion of agricultural activities, particularly coffee crops, seems to be a threat to the PNSL. During our fieldwork, we found that areas destined for this cultivation are being expanded between Arataca municipality and the PNSL borders. Within the PNSL, we noted the absence of monkey vocalizations and other mammal footprints on the trails and edges of streams. During the days in the field, although we did not hear shotguns, we did find some traps set up for hunting small mammals. Some residents have reported that hunting activity was frequent in the region. The areas of cabruca are still being utilized and we did not record any expansion of use during our visits. On one of the trails towards a mountain ridge, called “Peito de Moça” by locals, we saw an open area under recovery with abundant ferns and shrub vegetation and the presence of an abandoned wooden house. Among these threats, habitat loss was identified as the most visible and probably the main threat for amphibian and reptile species in Brazil (Rodrigues 2005; Silvano and Segalla 2005).

We conclude that the Parque Nacional da Serra das Lontras harbors a representative number of species of amphibians and reptiles, many of which are endemic to the Atlantic Forest and to the state. The new records of endemic, endangered, and species new to science reveal it as an outstanding area for the conservation and maintenance of ecological and evolutionary processes in this portion of southern Bahia, a region already known for its abundant biodiversity.

## Appendix I

Specimens deposited and examined in the herpetological collection of the Museu de Zoologia of the Universidade Estadual de Santa Cruz.

AMPHIBIA

**
Brachycephalidae
**

*Brachycephalus pulex* – MZUESC 21691–21697.

*Ischnocnema verrucosa* – MZUESC 21303, 21304, 21359, 21361, 21362.

Ischnocnema cf. parva – MZUESC 21306, 21393, 21404, 21405.

**
Bufonidae
**

*Rhinella crucifer* – MZUESC 21300, 21351, 21354, 21389, 21417, 21650, 21652, 21653.

**
Centrolenidae
**

*Vitreorana baliomma* – MZUESC 21037, 21039.

*Vitreorana eurygnatha* – MZUESC 21034, 21038, 21040, 21042, 21043, 21045.

*Vitreorana* sp. nov. – MZUESC 21044.

*Vitreorana uranoscopa* – MZUESC 21035, 21036, 21046.

**
Craugastoridae
**

“*Eleutherodactylus*” *bilineatus* – MZUESC 17025.

*Haddadus binotatus* – MZUESC 21298, 21309, 21385, 21387, 21390, 21392, 21394–21396, 21399–21401, 21403, 21408–21411, 21413, 21419, 21424, 21429, 21434.

*Pristimantis* sp. 1 – MZUESC 20995, 2009–21001, 21004, 21005, 21008, 21009, 21012, 21013, 21015, 21016, 21021, 21024, 21027, 21030, 21032, 21033.

*Pristimantis* sp. 2 – MZUESC 21443, 21454, 21482, 21495, 21496, 21513, 21535, 21550, 21559, 21577, 21580, 21584, 21588, 21590, 21591, 21594, 21604, 21610–21612, 21632, 21643, 21644, 21647.

*Pristimantis paulodutrai* – MZUESC 21447, 21485, 21486, 21492, 21497, 21507, 21538, 21539, 21541, 21593, 32452.

*Pristimantis vinhai* – MZUESC 21020, 21439–21441, 21448–21451, 21453, 21461, 21462, 21484, 21487–21491, 21494, 21494, 21508–21511, 21514–21518, 21525, 21526, 21528, 21531, 21532, 21537, 21540, 21549, 21551–21556, 21563–21567, 21571–21573, 21578, 21579, 21586, 21587, 21603, 21606, 21613, 21618–21622, 21641, 21642, 21648.

**
Eleutherodactylidae
**

*Adelophryne* sp. 2 – MZUESC 21445, 21446, 21502, 21504, 21505, 21519, 21522, 21529, 21575, 21583, 21602, 21638.

*Adelophryne* sp. 8 – MZUESC 21444, 21450, 21483, 21498, 21499, 21506, 21512, 21520, 21521, 21523, 21524, 21527, 21530, 21533, 21534, 21536, 21557, 21560, 21568–21570, 21576, 21585, 21589, 21596–21601, 21605, 21607–21609, 21615, 21616, 21623–21631, 21634–21637, 21639, 21645, 21646.

**
Hemiphractidae
**

*Gastrotheca recava* – MZUESC 21350, 21353, 21357, 21358.

**
Hylidae
**

*Aplastodiscus ibirapitanga* – MZUESC 21305.

*Aplastodiscus weygoldti* – MZUESC 21356.

*Boana crepitans* – MZUESC 2222, 2223.

*Boana faber* – MZUESC 21388, 21391, 21428, 21651, 21655, 21656.

*Boana pombali* – MZUESC 21397.

*Bokermannohyla lucianae* – MZUESC 21299, 21307, 21386, 21406, 21412, 21414, 21415, 21418, 21422, 21423, 21425–21427, 21430, 21431, 21437, 21438.

*Dendropsophus branneri* – MZUESC 21500, 21592.

Dendropsophus aff. bromeliaceus – MZUESC 21041, 21047.

*Dendropsophus elegans* – MZUESC 21558.

*Dendropsophus haddadi* – MZUESC 21456–21460, 21501, 21542–21548, 21581, 21582.

*Ololygon strigilata* – MZUESC 21352, 21402, 21407, 21416, 21433, 21435, 21436.

*Phyllodytes* sp. 1 – MZUESC 20994, 20996, 20997, 21000, 21002, 21003, 21006, 21007, 21010, 21011, 21014, 21017–21019, 21022, 21023, 21025, 21026, 21028, 21029, 21031, 21442, 21561, 21640.

Scinax cf. x-signatus – MZUESC 20408–20410, CFBH 44693.

**
Hylodidae
**

*Crossodactylus* sp. – MZUESC 20965–20971.

**Leptodactylidae**:

*Adenomera* clade M. – MZUESC 21713, 21714.

*Crossodactylodes septentrionalis* – MZUESC 14363, 21668.

Leptodactylus cf. latrans – MZUESC 21384, 21654.

**
Phyllomedusidae
**

*Phasmahyla spectabilis* – MZUESC 21301, 21360.

*Phyllomedusa burmeisteri* – MZUESC 21308, 21363.

REPTILIA

**
Amphisbaenidae
**

*Amphisbaena petrei* – MZUESC 16975.

*Leposternon* sp. – MZUESC 6707.

**
Boidae
**

*Corallus hortulanus* – MZUESC 1231, 1732, 3151, 3152, 6682.

*Epicrates cenchria* – MZUESC 2161, 4897, 8891.

**
Chelidae
**

*Hydromedusa maximiliani* – MZUESC 1235, 2189.

**
Colubridae
**

*Cercophis auratus* – MZUESC 1131.

*Chironius exoletus* – MZUESC 1102, 1122, 1228, 2167, 2236, 2237, 2904, 2905, 8861.

*Chironius foveatus* – MZUESC 1124, 8864.

*Chironius fuscus* – MZUESC 1101, 1003, 1125, 1130, 1137, 1138, 1220, 1744, 1755, 2234–2235, 6698, 6700.

*Chironius laevicollis* – MZUESC 6699.

*Coronelaps lepidus* – MZUESC 2227.

*Dipsas catesbyi* – MZUESC 21664, 2166, 4873.

*Dipsas indica* – MZUESC 1730, 4882.

*Dipsas neuwiedi* – MZUESC 1104–1106, 1127–1129, 1221–1223, 1232, 1233, 1736–1738, 1750, 2173, 2174, 21398, 2230–2233, 4272, 4425, 4493, 4874, 4875, 6687–6691, 6702, 8867.

*Dipsas variegata* – MZUESC 1108–1111, 1136, 1739, 1740, 2191, 2192, 4883, 6704, 6705.

*Drymoluber dichrous* – MZUESC 1528, 2247, 4881, 6683.

*Echinanthera cephalostriata* – MZUESC 1213.

*Elapomorphus wuchereri* – MZUESC 4489, 8890.

*Erythrolamprus aesculapii* – MZUESC 4876, 6692.

*Erythrolamprus miliaris* – MZUESC 2249.

*Erythrolamprus poecilogyrus* – MZUESC 2172.

*Erythrolamprus reginae* – MZUESC 1747, 1748, 21660, 2246, 2895, 6694.

*Erythrolamprus taeniogaster* – MZUESC 2901.

*Imantodes cenchoa* – MZUESC 1227, 19220, 21663.

*Leptodeira annulata* – MZUESC 1107, 1123, 1743, 2190, 2897, 4268, 4270, 4493, 4500, 6703.

*Oxybelis aeneus* – MZUESC 1224, 21662, 2171, 4427.

*Oxyrhopus formosus* – MZUESC 19221.

*Oxyrhopus guibei* – MZUESC 21665, 2226, 3791, 4878, 4879, 6706, 8887.

*Oxyrhopus petolarius* – MZUESC 1112, 1113, 1218, 1219, 1749, 2170, 2229, 2900, 4275, 4880.

*Philodryas olfersii* – MZUESC 8892.

*Pseudoboa nigra* – MZUESC 8862.

*Siphlophis compressus* – MZUESC 1234, 2168.

*Spilotes pullatus* – MZUESC 18800, 2164, 8881, 8882.

*Spilotes sulphureus* – MZUESC 2243, 3153, 4426, 4495, 4503, 4852, 8863.

Thamnodynastes cf. nattereri – MZUESC 19722, 2169, 2241, 2242, 2248, 4269, 4271, 4502, 6701.

*Xenodon rabdocephalus* – MZUESC 1133–1135, 1214, 1215, 1229, 1230, 1529, 1729, 1741, 1742, 2175–2179, 2193–2197, 2228, 2244, 2245, 2902, 2903, 2989, 3792, 4496–4999, 4273, 4274, 4424, 4884–4889, 6684–6686, 6696, 6697.

**
Dactyloidae
**

*Anolis fuscoauratus* – MZUESC 21420, 21421.

**
Elapidae
**

*Micrurus corallinus* – MZUESC 1746, 4877, 6693.

**
Gekkonidae
**

*Hemidactylus mabouia* – MZUESC 21355.

**
Gymnophtalmidae
**

*Leposoma nanodactylus* – MZUESC 21562, 21573, 21595, 21633.

*Leposoma scincoides* – MZUESC 21614.

**
Leiosauridae
**

*Enyalius catenatus* – MZUESC 1116, 1731, 2165, 21302, 21310, 21311, 21349, 21432, 21657–21659.

**
Polychrotidae
**

*Polychrus marmoratus* – MZUESC 1115, 1117.

**
Teiidae
**

*Ameiva ameiva* – MZUESC 1114, 1139–1140.

**
Tropidophiidae
**

*Tropidophis grapiuna* – MZUESC 19219.

**
Viperidae
**

*Bothrops bilineatus* – MZUESC 1119–1120, 1530, 21661, 2899, 3790, 4428–4430, 4869–4872, 6708.

*Bothrops jararaca* – MZUESC 1091–1100, 1121, 1126, 1132, 1216, 1225, 1226, 1727, 1728, 1733–1735, 2180–2188, 2198–2203, 3147–3150, 3787–3789, 4265–4267, 4501, 4417–4419, 4421–4423, 4431, 4490–4492, 6709–6721, 6695, 8865, 8866, 8868–8880, 8883–8886, 8888, 17480, 17822, 19719, 21666, 2238–2240, 4890–4896, 6670–6681.

*Bothrops leucurus* – MZUESC 1217, 2896, 4264, 4416, 4418, 4420.

*Lachesis muta* – MZUESC 2162, 2163, 4263, 4232–4434, 4504, 4898.
